# Inherited Disorders and Disease-Resistance Genomics in Kazakhstan Ruminants: Evidence, Limits and Breeding Priorities

**DOI:** 10.3390/ijms27146268

**Published:** 2026-07-14

**Authors:** Aizhan Mussayeva, Nurlan Malmakov, Berik Aringaziev, Kairly Omashev, Sholpan Bakhtybekkyzy, Aidana Bekitayeva, Lidiia Samarina

**Affiliations:** Institute of Genetics and Physiology, 050000 Almaty, Kazakhstan; nurlan_malmakov@mail.ru (N.M.); berik_aryngaziev@mail.ru (B.A.); okairly@mail.ru (K.O.); sholpan_bsb@mail.ru (S.B.); aidana.bekitayeva@gmail.com (A.B.)

**Keywords:** ruminant genomics, genomic health, inherited disorders, deleterious variants, disease resistance, carrier screening, cattle, sheep, goats, Kazakhstan

## Abstract

Kazakhstan ruminant genomics is expanding through targeted diagnostic testing, SNP-array studies, whole-genome sequencing, runs of homozygosity, candidate-gene analyses, transcriptomic studies and pathogen molecular diagnostics. However, these evidence types differ substantially in their relevance for breeding decisions. This structured narrative review evaluates molecular evidence for inherited disorders, deleterious alleles, disease-resistance loci, reproductive genes and genomic-health indicators in Kazakhstan cattle, sheep and goats. We define actionable evidence as evidence that can directly inform breeding management because it involves a validated pathogenic variant, risk variant or fertility haplotype detected or excluded in breeding-relevant animals or germplasm. Under this definition, cattle currently provide the strongest immediately actionable evidence, mainly because targeted studies have screened validated defects and fertility-related loci in artificial-insemination bulls, imported germplasm or breed-relevant populations. Evidence includes Kazakhstan-associated screening for BLAD (Bovine leukocyte adhesion deficiency), DUMPS (Deficiency of uridine monophosphate synthase), hypotrichosis, OH1-associated achromatopsia, fertility haplotypes and several beef- or dairy-breed recessive defects. In sheep, evidence is broader but less directly actionable, consisting mainly of prion protein gene preparedness, MHC (Major histocompatibility complex)-related immune hypotheses, reproductive candidate loci, runs of homozygosity, genome wide associated data and pathogen-exposure context. In goats, current evidence is mostly population-genomic and adaptation-oriented, while hereditary-disease surveillance and phenotype-linked resistance studies remain sparse. We propose an author-defined staged genomic-health framework that separates validated carrier-screening evidence from candidate genomic signals and international evidence requiring local validation. Priority actions include carrier-aware management of high-impact cattle germplasm, representative prion protein gene and runs of homozygosity baselines in small ruminants, phenotype-first surveillance, biobanking and national genotype–phenotype databases.

## 1. Introduction

Molecular livestock genetics in Kazakhstan is developing rapidly, but the available evidence remains fragmented across species, methods and levels of practical relevance. Recent studies have generated data from targeted diagnostic testing, SNP arrays, whole-genome sequencing, runs of homozygosity, GWAS, candidate-gene analyses, transcriptomic studies and pathogen molecular diagnostics in cattle, sheep and goats [1,2,3,4,5,6,7,8,9,10,11,12,13,14,15,16,17,18,19,20,21]. These data are highly valuable, but they do not all have the same breeding meaning. A validated pathogenic variant detected in an artificial-insemination bull can immediately inform mating decisions, whereas a ROH island, a selection signature near an immune gene, a pathogen PCR result or an unreplicated candidate association should usually be treated as hypothesis-generating evidence [22,23,24,25,26,27,28,29,30].

Kazakhstan is an important case for ruminant genomic-health analysis because large cattle, sheep and goat populations are managed under extensive continental environments while also being increasingly influenced by imported germplasm and modern genomic tools. National statistics report millions of cattle and sheep/goats, and the breeding landscape includes local, composite and internationally influenced resources such as Kazakh Whiteheaded, Alatau, Auliekol, Edilbay, Kazakh fat-tailed sheep, Kazakh meat-wool sheep, Arkhar-Merino and local goat ecotypes [1,2,3,4,5,6,7,8,9,10,11,12]. This diversity creates both opportunities and risks. Local populations may contain valuable adaptive variation, but adaptation does not imply freedom from deleterious alleles. Conversely, imported semen, embryos and breeding animals can accelerate genetic gain, but they can also introduce recessive defects or fertility-reducing haplotypes that remain hidden in heterozygous carriers [22,23,24,25,26,27,28,29,30].

The key scientific problem is therefore not simply to catalogue genes or breeds, but to determine which molecular findings are sufficiently validated for breeding action and which require further local validation. In cattle, Kazakhstan-specific evidence already includes screening for inherited defects and fertility-related loci, including BLAD, DUMPS, hypotrichosis-associated variants, OH1-associated achromatopsia variants, HH2, JH1, HH6, complex vertebral malformation, dilutor, osteopetrosis, arachnomelia, developmental duplication and arthrogryposis multiplex [31,32,33,34]. These studies are directly relevant to carrier-aware breeding because cattle breeding commonly uses artificial insemination and imported germplasm, allowing a single carrier sire to disseminate a pathogenic allele widely [22,23,24,25,26,27]. However, most available studies are still limited in sample size, breed representation and national prevalence inference.

In sheep and goats, the evidence base is different. Kazakhstan sheep studies provide important information on population diversity, reproductive candidate loci, PRNP preparedness, MHC-related immune hypotheses, GWAS and ROH patterns, but systematic evidence for confirmed hereditary disorders remains limited [35,36,37,38,39,40,41,42,43,44,45,46]. Goat evidence is still more preliminary: mtDNA, Y-chromosome and SNP-array studies show structured local ecotypes and candidate immune/adaptation regions, but direct hereditary-disease evidence and phenotype-linked disease-resistance validation are sparse [47,48,49,50]. In both species, candidate genes and pathogen-diagnostic data should not be interpreted as confirmed host-resistance evidence unless linked to animal genotype, phenotype and exposure context [42,45,46].

The main knowledge gap addressed by this review is the absence of a critical evidence framework for Kazakhstan ruminant genomic health. Existing studies are dispersed across different species, breeds, molecular methods and publication contexts, and they are often difficult to compare directly. It remains unclear which findings are immediately actionable for carrier management, which are useful mainly for surveillance prioritization, which require phenotype-linked validation and which are international findings relevant to Kazakhstan only through imported germplasm or ancestral breed components. This distinction is essential for avoiding both underuse of validated diagnostic tools and overinterpretation of candidate genomic signals.

This review therefore evaluates molecular evidence for inherited disorders, deleterious alleles, disease-resistance loci, reproductive genes, transcriptomic signals, pathogen-exposure context and genomic-health indicators in Kazakhstan cattle, sheep and goats. The objectives are as follows: (i) distinguish direct Kazakhstan evidence from comparative regional and international evidence; (ii) separate validated pathogenic or risk variants from candidate genomic signals; (iii) identify the main methodological and implementation limitations; and (iv) propose staged breeding and surveillance priorities that reduce avoidable genetic risk while conserving locally adapted ruminant resources.

## 2. Materials and Methods

### 2.1. Review Design

This article was prepared as a structured narrative review with evidence appraisal. A narrative rather than PRISMA-style systematic design was selected because the available evidence on Kazakhstan ruminant genomic health is heterogeneous in species, breed definitions, geographic origin, sampling strategy, molecular method, phenotype recording and practical breeding relevance. The aim was not to estimate pooled allele frequencies or effect sizes, but to critically classify published evidence according to its applicability for inherited-disorder surveillance, disease-resistance research, genomic-health monitoring and breeding implementation in cattle, sheep and goats.

The review addressed four questions: (i) which inherited disorders, pathogenic variants, carrier states or fertility-associated haplotypes have been reported or screened in Kazakhstan ruminants; (ii) which disease-resistance, immune, reproductive or adaptation loci are supported by direct evidence and which remain candidate-level; (iii) which studies are directly applicable to the Republic of Kazakhstan and which require local validation; and (iv) which actions are justified for breeding programs, surveillance and future research.

### 2.2. Literature Search and Source Identification

Targeted searches were conducted in PubMed/MEDLINE, Scopus, Web of Science, Google Scholar, AGRIS, eLIBRARY, CyberLeninka and accessible institutional repositories from Kazakhstan and neighboring regions. The main search covered publications from January 1991 to 10 June 2026. Earlier publications were retained only when they described validated causal mutations, internationally recognized fertility haplotypes, breed formation or genomic-health methods relevant to germplasm used in Kazakhstan [3,22,23,24,25,26,27,28,29,30,42,51,52,53,54,55,56,57,58,59].

Searches used four concept blocks. The first block defined geography and population: Kazakhstan, Kazakh, Kazakhstani, Central Asia, Kazakh Whiteheaded, Alatau, Auliekol, Aulieata, Edilbay, Kazakh fat-tailed sheep, Kazakh meat-wool sheep, Kazakh Arkhar-Merino, Baisary sheep and Kazakhstan local goats. The second block defined species: cattle, bovine, sheep, ovine, goat, caprine and ruminant. The third block defined genomic-health topics: inherited disorder, hereditary defect, genetic disease, deleterious variant, carrier screening, lethal haplotype, fertility haplotype, disease resistance, parasite resistance, mastitis resistance, scrapie, PRNP, MHC, GDF9, BMP15, BMPR1B, BLAD, DUMPS, CVM, hypotrichosis and OH1. The fourth block defined molecular methods: SNP, SNP array, WGS, whole-genome sequencing, GWAS, ROH, runs of homozygosity, transcriptome, RNA-seq, qPCR, PCR-RFLP, Sanger sequencing and pathogen genotyping. Searches were performed in English, Russian and Kazakh when relevant. The list of the abbleviations can be found in the end of the manuscript.

The final synthesis retained 87 sources. These included Kazakhstan-specific molecular or genomic studies, regional studies involving Kazakh-type populations, international studies validating pathogenic variants or fertility haplotypes relevant to imported germplasm, methodological papers on genomic-health interpretation, and selected pathogen-diagnostic studies used only as exposure context.

### 2.3. Eligibility Criteria

Sources were included when they met at least one of the following criteria: they studied cattle, sheep or goats bred in Kazakhstan; they reported molecular, genomic, veterinary-genetic or breeding data relevant to inherited disorders, carrier status, deleterious variants, fertility haplotypes, disease-resistance loci, immune-response genes, reproductive genes, ROH or genomic-health indicators; they described Kazakhstan breeds or breeding systems needed to interpret genetic risk; or they provided international validation for variants, haplotypes or methods relevant to breeds or germplasm used in Kazakhstan [60,61,62,63,64,65,66,67,68,69,70,71,72,73,74,75,76,77,78,79,80,81,82,83,84,85,86,87].

Sources were excluded from interpretive synthesis if they focused only on non-ruminant species, lacked a genetic or genomic component, duplicated another included dataset without adding relevant information, or described infectious disease without host-genetic relevance or exposure value. Molecular pathogen-diagnostic studies were retained only when they supplied Kazakhstan-specific exposure or phenotype context. They were not interpreted as evidence of host genetic resistance unless the same study also included host genotype, transcriptome, candidate loci or a breeding-relevant phenotype.

### 2.4. Evidence Appraisal

Each retained source was appraised using the following fields: species; breed or population; country and region of sampled animals; sample size; sampling frame; biological material; molecular method; tested gene, variant, haplotype or genomic region; phenotype definition; allele or genotype frequency when available; carrier status; genotype–phenotype linkage; and relevance for breeding or surveillance.

Geographic applicability was assessed explicitly. Studies conducted in the Republic of Kazakhstan were treated as direct Kazakhstan evidence. Studies involving Kazakh-named or Kazakh-type populations outside Kazakhstan, including some Xinjiang sheep studies, were treated as comparative regional evidence and not automatically generalized to Kazakhstan national populations. International studies were retained when they validated pathogenic variants, fertility haplotypes or genomic-health approaches relevant to imported semen, embryos, breeding animals or ancestral breed components used in Kazakhstan. To make this distinction explicit, all retained sources were additionally tagged in Supplementary Table S1 according to species, breed or population, country or region of sampled animals, evidence type, phenotype availability, genotype–phenotype linkage, applicability to Kazakhstan and evidence category. This table separates direct Kazakhstan evidence from comparative regional evidence, international germplasm-relevant evidence, pathogen-exposure evidence and methodological references.

### 2.5. Evidence Categories

Evidence was classified using a pragmatic four-level framework developed for this review (Table 1). This framework is not a formal clinical evidence-grading system and does not validate pathogenicity by itself. Its purpose is to prevent overinterpretation of heterogeneous molecular evidence.

Level A evidence was defined as Kazakhstan genotype–phenotype association or segregation evidence for a defined hereditary, disease-resistance, reproductive or survival phenotype. Level B evidence was defined as Kazakhstan detection or exclusion of a validated pathogenic variant, risk variant or fertility haplotype in breeding animals or germplasm. Level C evidence was defined as Kazakhstan SNP-array, WGS, ROH, GWAS, transcriptomic, candidate-gene or selection-signature evidence without direct disease confirmation. Level D evidence was defined as internationally validated disease variants, risk haplotypes or genomic-health methods relevant to breeds or germplasm used in Kazakhstan but not yet confirmed nationally [51,52,53,54,55,56,57].

Validated pathogenic variants detected in high-reproductive-impact animals were considered suitable for direct carrier management. Candidate immune, reproductive, adaptation or disease-resistance loci were considered priorities for phenotype-linked validation before marker-assisted selection [25,26,27,28,35,42,45,46].

### 2.6. Data Synthesis and Limitations

Evidence was synthesized by species and by genomic-health category. For cattle, the synthesis separated validated carrier-screening evidence, imported-germplasm risks, fertility haplotypes, ROH and population-genomic indicators, candidate health loci and pathogen-exposure context [60,61,65,67,68,70,71,72,73,74,75,76,77,78,79]. For sheep, the synthesis focused on PRNP preparedness, MHC-related immune hypotheses, parasite and infectious-disease resistance, reproductive loci, population-genomic structure and the gap in hereditary-disorder surveillance [62,63,64,66,69]. For goats, the synthesis focused on ecotype structure, SNP-array evidence, candidate immune/adaptation loci, PRNP preparedness and priorities for phenotype-first surveillance [12,19,42,44,47,48,49,50].

No meta-analysis was attempted because the studies differed substantially in species, breeds, molecular platforms, sample sizes, phenotype definitions, geographic origin and evidentiary strength. Negative carrier-screening results were interpreted only for the tested animals, tested variants and sampled lines, not as proof that a breed or national population is free from a disorder. Candidate genes, ROH islands, selection signatures and pathogen molecular diagnostics were treated as hypothesis-generating unless supported by host genotype–phenotype evidence. The review therefore emphasizes evidence strength, geographic applicability and breeding relevance rather than national prevalence estimates.

## 3. Kazakhstan Ruminant Genetic Resources

**Figure 1:**
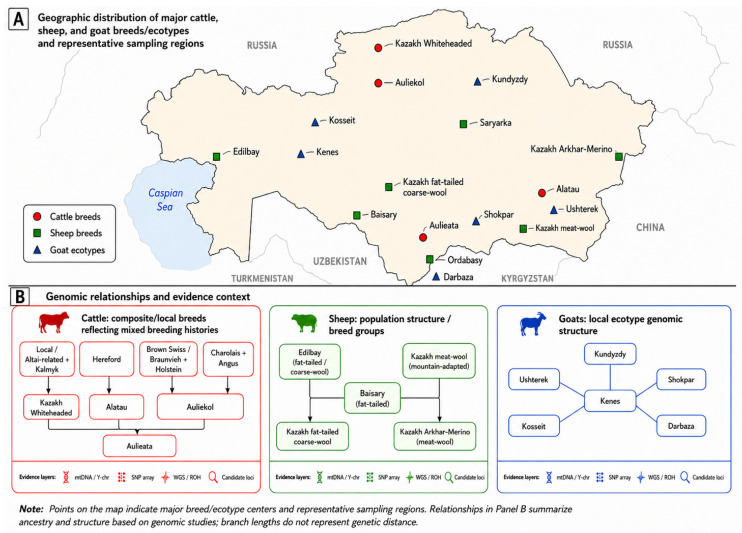
Geographic and genomic context of Kazakhstan ruminant resources. (**A**) Geographic distribution of major cattle breeds, sheep breeds and goat ecotypes, with representative sampling regions indicated by colored symbols. (**B**) Simplified schematic of genomic relationship context for cattle, sheep and goats, summarizing breed formation, population structure and local ecotype diversity. Evidence-layer icons indicate the main molecular data types considered: mtDNA/Y-chromosome markers, SNP arrays, WGS/ROH analyses and candidate loci. Branch lengths and connector positions are schematic and do not represent quantitative genetic distances.

Kazakhstan ruminant resources are best interpreted as genomic-health risk contexts rather than as descriptive breed categories. National cattle resources combine composite local breeds with imported dairy and beef germplasm. Kazakh Whiteheaded, Alatau and Auliekol cattle include Hereford, Brown Swiss/Braunvieh, Holstein, Charolais, Angus or other ancestral components; therefore, internationally validated cattle defects remain relevant even when animals are registered as Kazakhstan breeds (Figure 1, Table 2) [71,72,73,74,75,76,77,78,79]. Sheep resources are genetically diverse and regionally structured, but diversity alone does not demonstrate freedom from recessive defects or fertility risk [35,36,37,38,39,40,41,42,43,44]. Local goat ecotypes are structured and valuable for adaptation genomics, but direct hereditary-disease evidence remains sparse [12,19].

Earlier mtDNA, microsatellite, SNP-array, ROH and WGS studies are used here primarily to define ancestry, structure and sampling priorities. They establish that Kazakhstan cattle, sheep and goat resources are not homogeneous national categories, but they do not by themselves validate disease resistance or exclude hidden deleterious variation [12,14,15,16,17,18,19,20,21,28,29,30].

For genomic-health interpretation, three variables are more important than breed labels alone: ancestral components that define the relevant risk panel, reproductive impact of sampled animals, and quality of phenotype and pedigree documentation. The same variant has different management implications when it is detected in an imported semen batch, an AI bull, a community sire, an unrecorded village flock or a conservation herd [3,22,23,24,25,26,27,28,29,30,53,54,55,56,57].

In synthesis, the Kazakhstan resource base creates both opportunity and risk. Composite cattle ancestry and imported germplasm justify immediate attention to validated dairy- and beef-breed defects; structured sheep populations justify conservation and phenotype-linked validation; and goat ecotypes require phenotype-first surveillance before candidate immune or adaptation signals can be used for breeding [4,5,6,7,8,9,10,11,12,16,17,18,19,20,21,22,23,24,25,26,27,28,29,30,35,36,37,38,39,40,41,42,43,44,45,46,71,72,73,74,75,76,77,78,79].

The main unresolved issue is representativeness. Many studies provide first evidence from selected herds, breeds or germplasm sources, but they are not national prevalence surveys. Negative screening results should therefore be interpreted as absence of the tested variant in the tested animals, not as proof that a breed or population is free from the disorder. Future studies should report breed definition, sampling region, herd structure, reproductive role of sampled animals, pedigree depth and geographic applicability [28,29,30,53,54,55,56,57].

## 4. Cattle: Direct Carrier Evidence and the Imported-Germplasm Interface

Cattle provide the clearest immediate case for genomic-health control in Kazakhstan because validated recessive defects and fertility haplotypes can be screened in breeding animals with high reproductive impact. This does not imply that cattle have a higher genetic load than sheep or goats; rather, artificial insemination, imported semen and well-characterized international defect catalogs create a direct route from molecular diagnosis to mating control [22,23,24,25,26,27,71,72,73,74,75,76,77,78,79]. The strongest Kazakhstan-associated evidence is targeted carrier screening for DUMPS, BLAD, hypotrichosis and OH1-associated achromatopsia [25]. BLAD carriers detected among foreign-bred Holstein bulls represent actionable Category B evidence in the tested breeding material because the causal ITGB2/CD18 defect produces severe immunodeficiency in homozygous calves while carriers are clinically normal [25,73].

Other cattle findings also require evidence-specific interpretation. No DUMPS carriers were reported in the tested Holstein bulls, but because UMPS-associated DUMPS is expressed mainly through early embryonic mortality, negative results from limited samples should not remove DUMPS or other fertility-reducing loci from national panels [22,23,24,25,76,79]. Hypotrichosis-associated KRT71 carriers in Hereford and Angus samples and OH1-associated CNGB3 carriers in Alatau cattle support carrier-aware screening in breed-relevant germplasm, whereas absence in sampled Kazakh Whiteheaded cattle should be treated as sample-limited evidence rather than breed-wide freedom [25,31,75,78].

Population-genomic and candidate-gene studies provide a different type of evidence. WGS, SNP and ROH studies in Kazakh Whiteheaded, Alatau, Auliekol and related cattle identify ancestry, homozygosity and regions for future annotation, but these data are not direct evidence of pathogenicity [4,5,6,7,29,30,74]. HSP70 variation, ELOVL6/CRTC2 expression and SELL, MX1 or CXCR1 mastitis-associated loci are useful candidate or functional signals, but they require replicated genotype–phenotype validation, standardized health records and population-structure correction before routine selection use [45,46,60,67,77].

Pathogen molecular diagnostics should also be separated from host resistance evidence. Studies of Anaplasma spp., bovine leukemia virus, piroplasms, Ehrlichia and Theileria in Kazakhstan cattle define exposure landscapes and diagnostic infrastructure, but they do not demonstrate host genetic resistance unless paired with host genotype and comparable clinical outcomes [61,64,65,68,70].

Thus, cattle evidence separates into two practical classes (Table 3). Validated monogenic defects and fertility haplotypes such as BLAD, DUMPS, hypotrichosis, OH1, CVM, brachyspina, arachnomelia, developmental duplication and arthrogryposis multiplex justify carrier-aware mating and pre-use certification [25,31,32,33,34,71,72,73,74,75,76,77,78]. Polygenic traits such as mastitis resistance, heat tolerance, immune competence, fertility and longevity require replicated phenotype-linked studies before marker-assisted selection [45,46,60,67,77].

Overall, cattle currently provide the most immediately actionable component of Kazakhstan ruminant genomic health because validated defects and fertility haplotypes can be connected directly to AI-bull testing, semen certification and mating control [22,23,24,25,26,27,31,32,33,34,71,72,73,74,75,76,77,78,79]. Detection of BLAD carriers among foreign-bred Holstein bulls, OH1-associated carriers in Alatau cattle and hypotrichosis-associated carriers in Hereford or Angus backgrounds should therefore be interpreted as Category B evidence suitable for carrier-aware management in the tested breeding material [25,31,34,73,75,78].

However, the current cattle evidence should not be overextended. Most Kazakhstan studies are targeted diagnostic studies or population-genomic analyses with limited sample sizes, selected breed representation, or incomplete reproductive records. They are sufficient to justify surveillance and pre-use certification, but insufficient to estimate national carrier prevalence or to conclude that unsampled lines are free from defects. This limitation is particularly important for fertility haplotypes and embryo-lethal variants because their effects are often expressed as repeat insemination, early embryonic loss, prolonged calving interval or absence of homozygotes rather than as visible affected calves [22,23,24,32,33,76,79]. For such defects, molecular screening must be linked to insemination, pregnancy, calving, pedigree and semen-distribution records.

The cattle evidence also illustrates a broader methodological point: monogenic carrier control and polygenic disease-resistance selection require different standards of proof. For BLAD, DUMPS, OH1, hypotrichosis, CVM, brachyspina, arachnomelia, developmental duplication and arthrogryposis multiplex, the immediate breeding objective is to avoid carrier-by-carrier matings while preserving useful genetics [25,31,32,33,34,71,72,73,74,75,76,77,78]. By contrast, mastitis resistance, fertility, heat tolerance, immune competence and longevity are complex traits that require replicated genotype–phenotype associations, standardized phenotypes, population-structure correction and evaluation against broader breeding goals before routine marker-assisted selection is justified [45,46,60,67,77]. Therefore, the immediate priority for Kazakhstan cattle is not indiscriminate exclusion of all carriers, but systematic testing of high-impact animals, transparent variant nomenclature, integration of carrier status into mating plans and ROH-based monitoring of inbreeding in nucleus and composite populations [26,27,28,29,30,58,59,74].

## 5. Sheep: Disease Resistance, Reproductive Loci and a Hereditary-Disorder Gap

Sheep evidence in Kazakhstan is biologically broad but less immediately actionable than cattle evidence. Microsatellite, mtDNA, OvineSNP50, ROH and GWAS studies demonstrate diversity, farm or breed structure and production-related genomic signals in Edilbay, Kazakh fat-tailed, Kazakh fine-wool, Baisary and related populations [8,9,10,11,16,17,18,19,20,21]. These data are important for conservation and sampling design, but they should not be interpreted as hereditary-disease surveillance or validated resistance evidence. High diversity can coexist with local founder effects, line-specific recessive alleles and unrecorded neonatal or reproductive losses [28,29,30,53,54,55,56].

PRNP is the most immediate disease-susceptibility locus for sheep. Internationally, codons 136, 154 and 171 define major scrapie risk categories, with ARR generally associated with resistance and VRQ with high susceptibility [43,44]. For Kazakhstan, the key point is not that scrapie is established as a national problem; it is that a disease with a validated host-genetic component can be managed only if baseline PRNP frequencies are known. Absence of reported scrapie cases should therefore be treated as an incomplete surveillance state, not as evidence that susceptibility alleles are absent.

MHC class II genes, particularly DRB1 and DQB1, should be presented as biologically plausible candidate loci rather than validated selection markers for Kazakhstan sheep. Their role in antigen presentation supports further work, but associations with echinococcosis, gastrointestinal parasites or other infections can be confounded by breed structure, exposure, diagnostic method, vaccination and management [35,42,45,46]. For breeding use, MHC data require standardized phenotypes such as fecal egg count, hydatid cyst burden, slaughterhouse lesions, serology, survival, body condition and lamb growth.

Transcriptomic and pathogen-diagnostic studies should be interpreted as context for future host-genetic validation. A comparative spleen transcriptome study of indigenous Kazakh sheep and Suffolk sheep in Xinjiang identified immune-responsive genes and pathways relevant to adaptation and host defense [66]. PCR or sequencing studies of Anaplasma ovis, Coenurus cerebralis, piroplasms, Ehrlichia and sheeppox virus provide exposure and phenotype context; they become host-resistance evidence only when linked to host genotype, standardized phenotype and comparable exposure conditions [62,63,64,69].

Reproductive loci also require cautious translation. GDF9 and BMP15 variation has been reported in Kazakh meat–wool sheep, and prolificacy-related selection signatures have been described in Chinese and Kazakhstan sheep breeds [36,39]. International evidence shows that BMP15, GDF9 and BMPR1B variants can affect ovulation rate and litter size, but some alleles have genotype-dependent fertility costs [37,38,40,41]. Therefore, fecundity genotypes should be evaluated together with litter size, lamb survival, ewe fertility, longevity, nutrition and lambing-management capacity.

In synthesis, Kazakhstan sheep evidence remains mainly Category C evidence, with PRNP as the clearest internationally validated preparedness locus. PRNP genotyping can support risk-management baselines, but broad selection against risk genotypes should be balanced against conservation of locally adapted diversity and supported by Kazakhstan-specific surveillance [42,43,44].

For MHC loci, parasite-resistance markers, transcriptomic signals and immune-response candidates, the current role is prioritization rather than implementation. These markers can guide selection of flocks, pathogen systems and candidate haplotypes for deeper validation, but they should not replace field phenotyping or genomic prediction based on standardized exposure and outcome data [35,42,45,46,62,63,64,66,69].

Reproductive loci such as GDF9, BMP15 and BMPR1B require similarly cautious interpretation. These genes are strong biological candidates, and major-effect variants in these pathways have well-established effects on ovulation rate or prolificacy in several sheep populations [36,37,38,39,40,41]. However, increased ovulation rate is not automatically equivalent to improved breeding value in low-input or extensive systems. Without adequate nutrition, lambing supervision, veterinary support and selection for lamb survival, prolificacy alleles can increase dystocia, triplet mortality, ewe metabolic stress or management costs. For Kazakhstan sheep, the appropriate next step is not direct selection on candidate genotypes alone, but validation against litter size, lamb survival, ewe longevity, barren-ewe rate and flock-level productivity. Thus, the main scientific opportunity in Kazakhstan sheep is to move from candidate markers to integrated genotype–phenotype–environment datasets that reflect real grazing systems.

## 6. Goats: Valuable Adaptation Genomics, Sparse Disease Genetics

Goat evidence is currently population-genomic and adaptation-oriented rather than disease-genetic. The 70K SNP study of Kundyzdy, Darbaza, Shokpar, Ushterek, Kenes and Kosseit ecotypes demonstrates structure, diversity, ROH patterns and selection signatures in Kazakhstan local goats [12]. Immune and adaptation candidates such as NLRC4, HCLS1, IL17D, IL17RE and IL17RC are biologically plausible pathway signals, but they remain Category C evidence: selection signatures near immune genes do not prove resistance to parasites, respiratory disease, mastitis, brucellosis exposure or kid mortality [12,42,45,46].

The international goat literature identifies priorities that should inform Kazakhstan surveillance but not be assumed present nationally. PRNP codons including 146 and 222 are relevant to scrapie preparedness, while polled intersex syndrome and beta-mannosidosis illustrate how recessive or genotype-dependent disorders can persist when abnormal phenotypes are poorly recorded [44,47,48,49,50]. Kazakhstan-specific evidence for these conditions remains sparse and should be described as an evidence gap rather than evidence of absence.

In synthesis, Kazakhstan goats are the least developed component of the national ruminant genomic-health agenda. Available mtDNA, Y-chromosome and SNP-array data define ancestry, ecotype structure and candidate adaptation regions, but they do not establish hereditary-disease prevalence or validated resistance markers [12,19]. Candidate immune genes should therefore be treated as pathway-level hypotheses rather than direct selection targets [12,42,45,46]. The immediate priority in goats is phenotype-first surveillance. Household management, informal buck exchange, limited pedigree recording and low diagnostic capture can hide congenital defects, intersex phenotypes, kid mortality, neurological syndromes, infertility, udder disease, respiratory disease and parasite susceptibility [48,49,50]. Future work should combine WGS of representative ecotypes, breeding bucks and affected families with standardized recording of kid survival, reproductive abnormalities, parasite burden, mastitis, respiratory disease and growth under grazing [12,28,29,30,42,45,46,53,54,55,56,57].

## 7. Cross-Species Critical Synthesis and Unresolved Questions

Across cattle, sheep and goats, the central issue is not the absence of molecular data, but the uneven evidentiary value of different data types. Kazakhstan cattle already have the strongest basis for immediate genomic-health action because several studies target validated recessive defects, fertility haplotypes or internationally recognized pathogenic variants in breeding-relevant animals [25,31,32,33,34,71,72,73,74,75,76,77,78,79]. Sheep evidence is stronger for disease-resistance hypotheses, reproductive candidate loci and adaptive population structure, but weaker for confirmed hereditary-disorder surveillance [8,9,10,11,16,17,18,20,21,35,36,37,38,39,40,41,42,43,44,45,46,62,63,64,66,69]. Goat evidence is strongest for population structure and adaptation genomics, while direct hereditary-disease and phenotype-linked resistance data remain sparse [12,19,44,47,48,49,50]. Therefore, a single implementation model cannot be applied uniformly across species.

The first unresolved question is how to distinguish national prevalence from local or selected-sample evidence. Most current studies are not designed as representative national surveys. This is acceptable for initial discovery or method development, but it limits inference. For carrier control, testing all high-impact breeding males, semen batches and nucleus animals is more important than estimating population prevalence precisely. For national risk estimation, however, sampling must be stratified by breed, region, herd, sire line and germplasm origin, with confidence intervals reported for allele frequencies. Negative results from small samples should be described only as negative for the tested animals and tested variants, not as evidence that a breed is free from the disorder [26,27,28,29,30,53,54,55,56,57].

For practical surveillance, sample-size interpretation should be linked to the objective of testing. If the objective is to prevent dissemination of known recessive variants through high-impact breeding animals, sampling is not appropriate: all artificial-insemination bulls, imported semen batches, embryos, elite sires, nucleus animals and major breeding males should be tested. If the objective is to detect whether a validated carrier state is present above a defined frequency in a breed or line, binomial detection logic can be used. For example, to have approximately 95% probability of detecting at least one carrier, assuming random sampling within a stratum, about 29 animals are required when carrier frequency is 10%, 59 animals when carrier frequency is 5%, 149 animals when carrier frequency is 2% and 299 animals when carrier frequency is 1%. These numbers should be interpreted as minimum values per relevant stratum, not as national sample sizes, because Kazakhstan ruminant populations are structured by breed, region, herd, sire family and germplasm origin [26,27,28,29,30,53,54,55,56,57,87]. For prevalence estimation rather than simple detection, larger samples are required and confidence intervals should be reported. For example, an approximate binomial estimate with ±5% precision requires about 384 animals under the most conservative assumption, whereas rare carrier-frequency estimation may require several hundred to several thousand animals depending on the expected frequency and desired precision. Therefore, small negative studies are useful for the tested animals but should not be used to declare national freedom from a variant.

The second unresolved question is how to connect host genomics with disease exposure. Pathogen PCR, qPCR, sequencing or phylogenetic analysis provides valuable exposure information, but it is not evidence of host genetic resistance unless linked to animal genotype, standardized phenotype and comparable exposure conditions [35,42,45,46,61,62,63,64,65,68,69,70]. Future disease-resistance studies should therefore be designed around paired host–pathogen datasets. For example, studies of tick-borne disease, sheeppox, BLV, Anaplasma, Theileria or parasite resistance should record pathogen species or strain, diagnostic method, vaccination status, clinical outcome, management system and host genotype in the same animals. Without this structure, apparent resistance markers may reflect differences in exposure, breed composition or management rather than inherited resistance.

The third unresolved question is how to manage deleterious alleles without losing adaptive diversity. Carrier exclusion is straightforward when a widely used AI bull carries a severe recessive allele and alternative non-carrier genetics are available. It is more complex in small local breeds, conservation herds, or goat ecotypes where a carrier may also represent rare adaptive ancestry. In such cases, carrier-aware mating, avoidance of carrier-by-carrier matings, and gradual allele-frequency reduction are preferable to indiscriminate culling [26,27,28,29,30,55]. Genomic-health programs should therefore combine targeted diagnostics with ROH monitoring, effective population-size management and conservation of local adaptive variation [28,29,30,55,58,59,74].

The fourth unresolved question is how to build local analytical capacity. Genomic-health implementation requires more than laboratory genotyping. It requires local pipelines for WGS quality control, SNP-array processing, imputation, ROH analysis, haplotype detection, variant-effect prediction, pathogenicity classification and integration with pedigree and phenotype records [80,81,82,83,84,85,86,87]. Kazakhstan data should also be made interoperable with international resources such as OMIA and the 1000 Bull Genomes framework where data governance allows, because this would improve variant interpretation, facilitate recognition of private or locally enriched variants and reduce duplication of discovery efforts [51,52,53,54,55,56,57]. At the same time, international databases cannot substitute for Kazakhstan-specific phenotype validation, especially in local breeds and low-input production systems.

The practical implication is that Kazakhstan should use a tiered strategy. The first tier is immediate carrier control for validated cattle defects and high-impact breeding animals. The second tier is baseline surveillance for PRNP, ROH and major reproductive or hereditary-risk loci in sheep and goats. The third tier is phenotype-first discovery of local defects through systematic recording, biobanking and WGS of affected animals and relatives. The fourth tier is development of national genotype–phenotype databases that connect molecular results with reproduction, survival, disease, production and conservation outcomes. This staged approach avoids both underuse of available diagnostic tools and overinterpretation of candidate genomic signals.

## 8. Breeding Implementation and Research Priorities

Implementation should follow the evidence hierarchy summarized in Table 4 rather than a single technology-first model. The first priority is targeted testing of high-reproductive-impact animals: AI bulls, imported semen, embryos, elite cattle sires and nucleus-herd dams, and breeding rams or bucks in pedigree, nucleus or community-mating systems [25,31,32,33,34,71,72,73,75,76,78]. The second priority is genomic-health monitoring through SNP arrays, ROH analysis and haplotyping, with carrier-aware mating rather than indiscriminate culling in small or locally adapted populations [26,27,28,29,30,55,58,59,74]. The third priority is phenotype-first surveillance and biobanking for congenital defects, abortions, stillbirths, neonatal mortality, recurrent infections, ocular defects, hair or wool abnormalities, limb malformations, intersex phenotypes and unexplained infertility [53,54,55,56].

The economic rationale for this staged approach is strongest where genetic defects cause invisible losses. Fertility haplotypes, DUMPS and other embryo-lethal or prenatal-loss variants may appear as repeat insemination, prolonged calving interval, early embryonic loss or absence of expected homozygotes rather than as recognizable affected offspring [22,23,24,32,33,76,79]. Conversely, severe recessive defects detected in high-use breeding animals can be managed cost-effectively by pre-use testing and avoiding carrier-by-carrier matings [25,26,31,32,33,34,71,72,73,74,75,76,77,78]. For sheep and goats, the implementation priority is not immediate selection on candidate genes, but PRNP baselines, ROH monitoring, phenotype-linked validation of reproductive or immune candidates, and WGS of affected families [12,35,36,37,38,39,40,41,42,43,44,45,46,47,48,49,50,53,54,55,56,57]. National databases should tag each record by evidence type, geographic applicability, phenotype quality and genotype–phenotype linkage; local pipelines for WGS quality control, imputation, ROH analysis, haplotype detection, variant-effect prediction and pathogenicity classification are required for this system to become operational [51,52,53,54,55,56,57,80,81,82,83,84,85,86,87].

## 9. Conclusions

Kazakhstan ruminant genomics has reached the stage where the main challenge is no longer simply to generate molecular data, but to interpret heterogeneous evidence for breeding, surveillance and conservation. The current evidence base is uneven across species. Cattle provide the strongest immediate case for genomic-health implementation because validated recessive defects and fertility haplotypes can be screened in high-impact breeding animals, imported semen, embryos and nucleus herds. However, even in cattle, most Kazakhstan studies are too limited to estimate national prevalence, and negative results should not be generalized beyond the tested animals and variants [25,31,32,33,34,71,72,73,74,75,76,77,78,79].

Sheep evidence is broader but less directly actionable. Population-genomic studies show valuable diversity and structure, and candidate loci such as PRNP, MHC-region genes, GDF9, BMP15 and BMPR1B are relevant to disease preparedness, immune response and reproduction [8,9,10,11,16,17,18,20,21,35,36,37,38,39,40,41,42,43,44,45,46]. Nevertheless, most sheep findings require phenotype-linked validation before routine marker-assisted selection. The highest priorities are representative PRNP baselines, systematic recording of congenital and reproductive abnormalities and integrated studies linking host genotype with parasite burden, pathogen exposure, lamb survival, ewe fertility and productivity under real grazing conditions.

Goats remain the largest evidence gap. Current data demonstrate that Kazakhstan local goat ecotypes are structured and genetically valuable, but direct evidence for hereditary disorders, validated disease-resistance loci and national carrier risks is sparse [12,19,44,47,48,49,50]. The priority for goats is therefore phenotype-first surveillance, WGS of representative ecotypes and affected families, baseline PRNP genotyping, ROH monitoring, and systematic recording of kid survival, reproductive anomalies, udder health, respiratory disease, parasite burden and growth.

Across species, genomic-health implementation should follow a staged model. Validated pathogenic variants in high-impact breeding animals should be managed immediately through testing and carrier-aware mating. Candidate genes, ROH islands, selection signatures, transcriptomic signals and pathogen molecular diagnostics should be used to prioritize surveillance and hypothesis testing, but not treated as confirmed resistance or disease markers without phenotype-linked validation. National progress will depend on biobanking, standardized variant nomenclature, interoperable genotype–phenotype databases, local bioinformatics capacity and integration with international resources such as OMIA and global cattle genome datasets [51,52,53,54,55,56,57,80,81,82,83,84,85,86,87]. The long-term goal should be a balanced genomic-health strategy that reduces avoidable hereditary disease and fertility loss while conserving the adaptive diversity of Kazakhstan cattle, sheep and goats.

## Figures and Tables

**Table 1:** Pragmatic evidence categories used for interpreting Kazakhstan ruminant genomic-health findings. AI, artificial insemination; GWAS, genome-wide association study; OMIA, Online Mendelian Inheritance in Animals; ROH, runs of homozygosity; SNP, single nucleotide polymorphism; WGS, whole-genome sequencing. Categories A–D are used as interpretive categories for this review and should not be read as a validated hierarchy of clinical certainty. In practice, a Category B finding in a high-use AI bull may be more immediately actionable for breeding than a Category A association from a small, unreplicated dataset. Conversely, Category C evidence may be scientifically important but requires phenotype-linked validation before implementation.

Category	Evidence Type	Appropriate Interpretation	Main Limitation	Example in This Review
A. Direct Kazakhstan genotype–phenotype evidence	Genotype–phenotype association, segregation evidence, or WGS-based variant discovery in Kazakhstan animals with a defined hereditary, reproductive, disease-resistance, survival or production phenotype.	Can support strong local inference when phenotype definition, sampling, pedigree or family structure and statistical analysis are adequate. May justify validation studies, targeted surveillance or breeding decisions in the studied population.	Does not automatically establish national prevalence or applicability to all breeds, regions or management systems. Requires replication and careful control for population structure and environmental exposure.	Future WGS of affected calves, lambs or kids with parental genotypes; phenotype-linked host-genetic studies of parasite resistance, fertility or survival.
B. Kazakhstan carrier-screening or diagnostic evidence for validated variants	Detection or exclusion of internationally validated pathogenic variants, risk variants or fertility haplotypes in Kazakhstan breeding animals, imported germplasm or breed-relevant populations.	Directly useful for carrier-aware mating, pre-use certification of AI bulls, semen, embryos, nucleus animals or high-impact breeding males.	Negative results apply only to the tested animals, variants and sampled lines. They do not prove that a breed or national population is free from the disorder.	BLAD carriers in foreign-bred Holstein bulls; OH1-associated carriers in Alatau cattle; hypotrichosis-associated variants in Hereford/Angus-related cattle [25,31,34,73,75,78].
C. Kazakhstan candidate genomic, transcriptomic or population-genomic evidence	SNP-array, WGS, ROH, GWAS, transcriptomic, candidate-gene, selection-signature, diversity or adaptation evidence from Kazakhstan animals without direct confirmation of disease causality or validated resistance effect.	Useful for hypothesis generation, surveillance prioritization, identification of populations for deeper sampling and design of phenotype-linked validation studies.	Should not be used alone for routine marker-assisted selection for disease resistance, fertility, adaptation or hereditary-disease control. Candidate signals may reflect ancestry, population structure, drift, selection history or environmental confounding.	ROH and population-genomic studies in Kazakh cattle and sheep; immune/adaptation candidate genes in Kazakhstan local goats; candidate reproductive loci in sheep [4,5,6,7,8,9,10,11,12,16,17,18,19,20,21,35,36,37,38,39,60,66,67,74].
D. International evidence relevant to Kazakhstan germplasm or methods	Internationally validated pathogenic variants, fertility haplotypes, disease-resistance loci, genomic-health methods, OMIA records, 1000 Bull Genomes evidence or livestock-genomic frameworks relevant to breeds, ancestral components or imported germplasm used in Kazakhstan.	Supports design of national diagnostic panels, surveillance programs, variant-prioritization schemes and interpretation of imported-germplasm risk.	Does not prove that the variant, haplotype or risk allele is present in Kazakhstan. Requires local screening and, where relevant, Kazakhstan-specific phenotype validation.	Holstein lethal/fertility haplotypes; CVM; brachyspina; PRNP risk categories; OMIA and WGS-based mutation-discovery frameworks [22,23,24,43,44,51,52,53,54,55,56,57,71,72,79].

**Table 2:** Species-specific resources and genetic-health interpretation.

Species/Resource	Breeding Context	Best Current Molecular Evidence	Main Genetic-Health Interpretation	Immediate Priority
Kazakh Whiteheaded cattle	National beef composite adapted to extensive grazing	WGS, resequencing, ancestry and selection-signature studies [4,5,6]	Useful adaptation background; risk panel should include beef-breed defects because of Hereford and other ancestry	AI-bull screening, ROH monitoring, representative WGS
Alatau cattle	Dairy/dual-purpose composite with Brown Swiss/Braunvieh and Holstein influence	SNP studies and OH1 carrier screening [4,7,25,75]	Composite dairy ancestry makes internationally known recessive defects relevant	Screen OH1 and broader dairy-defect panels
Auliekol cattle	Modern beef composite involving Kazakh Whiteheaded, Charolais and Angus components	SNP and ROH evidence [7,74]	Genomic inbreeding and imported beef-line defects require monitoring	Broad beef panel plus genomic inbreeding control
Holsteinized dairy cattle	Imported semen and elite dairy breeding	BLAD and DUMPS screening; mastitis-candidate studies [25,73,76,77]	Highest immediate carrier-screening priority	Mandatory pre-use semen and AI-bull certification
Edilbay and fat-tailed sheep	Low-input grazing, meat/fat-tail adaptation	Microsatellite, SNP, mtDNA, OvineSNP50 and ROH studies [8,9,10,11,16,20,21]	Diversity is high but not equivalent to disease freedom	PRNP baseline, parasite-resistance phenotypes, WGS of abnormal cases
Kazakh meat-wool sheep	Meat–wool production and fertility improvement	GDF9/BMP15 variation [36,37,38]	Reproductive loci require phenotype-linked validation	Litter size, ewe fertility and lamb survival datasets
Local goat ecotypes	Household and regional adaptation	70K SNP diversity, ROH and immune/adaptation candidates [12]	Promising adaptation resource but hereditary-disease evidence is sparse	Phenotype-first surveillance, PRNP baseline, WGS

**Table 3:** Priority cattle defects, loci and genomic-health indicators for Kazakhstan.

Target	Gene/Locus	Breed Relevance	Kazakhstan Evidence	Breeding Action
BLAD	ITGB2/CD18	Holstein and Holsteinized dairy cattle	Carriers detected among foreign-bred Holstein bulls [25,73]	Mandatory artificial-insemination bull and semen screening; avoid carrier matings
DUMPS	UMPS	Holstein-derived dairy germplasm	No carriers in tested bulls, but sample-limited [25,76]	Keep in dairy panel because phenotype is hidden embryo loss
Hypotrichosis (HY)	KRT71	Hereford, Angus, Kazakh Whiteheaded and Auliekol risk context	Carriers detected in tested Hereford/Angus; absent in sampled Kazakh Whiteheaded [25,78]	Screen imported beef germplasm and representative composite lines
OH1 achromatopsia	CNGB3	Alatau and Brown Swiss/Braunvieh-related cattle	Carriers detected in Alatau cattle [25,75]	Include in Alatau and related dairy/dual-purpose panels
CVM, brachyspina, citrullinemia, factor XI deficiency, cholesterol deficiency	SLC35A3, FANCI, ASS1, F11, APOB and others	Holstein, Brown Swiss and other dairy germplasm	No robust Kazakhstan prevalence data identified	Screen elite dairy sires, imported semen and embryos [71,72]
Holstein lethal/fertility haplotypes	HH1, HH2, HH3, HH5, HH6, JH1 and related haplotypes	Holsteinized dairy populations	HH2, JH1 and HH6 diagnostic evidence exists in Holstein/Jersey or Holstein cow studies; broader national prevalence remains unresolved [32,33]	Use diagnostic haplotyping with reproductive records [22,23,24,32,33,79]
Genomic inbreeding and ROH	Genome-wide	Kazakh Whiteheaded, Auliekol, Alatau and nucleus herds	Kazakhstan SNP studies report ROH patterns [74]	Monitor effective population size and avoid excessive sire concentration

**Table 4:** Staged genomic-health roadmap for Kazakhstan ruminants.

Stage	Main Objective	Cattle Emphasis	Sheep/Goat Emphasis	Expected Output
1. Immediate diagnostics	Prevent known carrier risk in high-impact animals	Mandatory artificial-insemination bull, semen and embryo panels	PRNP baseline in breeding males; targeted tests where relevant	Carrier certificates and mating restrictions
2. Genomic-health monitoring	Control inbreeding and hidden fertility haplotypes	SNP arrays, ROH and haplotype analyses in nucleus herds	ROH and diversity monitoring in local breeds/ecotypes	Breed-specific risk dashboards
3. Phenotype-first surveillance	Discover local defects and validate candidate markers	Affected calf/family WGS and reproductive-record linkage	Affected lamb/kid WGS; parasite, fertility and survival phenotypes	Genotype–phenotype case series and validated associations
4. Reference populations	Estimate breeding values for health and resilience	Mastitis, fertility, survival and disease-resistance records	Parasite burden, lamb/kid survival, reproductive efficiency	Genomic prediction for health traits
5. Conservation integration	Preserve adaptive diversity while reducing deleterious load	Local breed WGS panels and controlled introgression	Community and breed-conservation sampling	Balanced selection and conservation decisions

## Data Availability

No new datasets were generated or analyzed in this review. All sources are cited in the reference list.

## Supplementary Materials

The following supporting information can be downloaded at: https://www.mdpi.com/article/10.3390/ijms27146268/s1.

## Abbreviations

**Abbreviation**	**Full Term**
AGRIS	International System for Agricultural Science and Technology
AI	Artificial insemination
APOB	Apolipoprotein B
ARR	Alanine–arginine–arginine PRNP haplotype
ASS1	Argininosuccinate synthase 1
BLAD	Bovine leukocyte adhesion deficiency
BLV	Bovine leukemia virus
BMP15	Bone morphogenetic protein 15
BMPR1B	Bone morphogenetic protein receptor type 1B
CAST	Calpastatin
CD18	Cluster of differentiation 18; integrin beta-2
CNGB3	Cyclic nucleotide-gated channel beta 3
CRTC2	CREB-regulated transcription coactivator 2
CVM	Complex vertebral malformation
CXCR1	C-X-C motif chemokine receptor 1
D-loop	Mitochondrial displacement loop
DNA	Deoxyribonucleic acid
DQB1	Major histocompatibility complex class II DQ beta 1
DRB1	Major histocompatibility complex class II DR beta 1
DUMPS	Deficiency of uridine monophosphate synthase
ELOVL6	ELOVL fatty acid elongase 6
F11	Coagulation factor XI
FANCI	Fanconi anemia complementation group I
FAT1	FAT atypical cadherin 1
FZD3	Frizzled class receptor 3
GDF9	Growth differentiation factor 9
GWAS	Genome-wide association study
HCLS1	Hematopoietic cell-specific Lyn substrate 1
HH1	Holstein haplotype 1
HH2	Holstein haplotype 2
HH3	Holstein haplotype 3
HH5	Holstein haplotype 5
HH6	Holstein haplotype 6
HSP70	Heat-shock protein 70
HY	Hypotrichosis
IL8	Interleukin 8
IL17A	Interleukin 17A
IL17D	Interleukin 17D
IL17RC	Interleukin 17 receptor C
IL17RE	Interleukin 17 receptor E
ITGB2	Integrin subunit beta 2
JH1	Jersey haplotype 1
KRT71	Keratin 71
MEDLINE	Medical Literature Analysis and Retrieval System Online
MHC	Major histocompatibility complex
mRNA	Messenger RNA
MSTN	Myostatin
mtDNA	Mitochondrial DNA
MX1	MX dynamin-like GTPase 1; myxovirus resistance protein 1
NLRC4	NLR family CARD domain-containing protein 4
OH1	OH1-associated recessive achromatopsia haplotype/variant in Original Braunvieh/Brown Swiss-related cattle
OMIA	Online Mendelian Inheritance in Animals
OvineSNP50	Ovine 50K SNP genotyping array
PCR	Polymerase chain reaction
PCR-RFLP	Polymerase chain reaction–restriction fragment length polymorphism
PRISMA	Preferred Reporting Items for Systematic Reviews and Meta-Analyses
PRNP	Prion protein gene
qPCR	Quantitative polymerase chain reaction
RNA	Ribonucleic acid
RNA-seq	RNA sequencing
ROH	Runs of homozygosity
SELL	Selectin L
SLC35A3	Solute carrier family 35 member A3
SNP	Single nucleotide polymorphism
SNP array	Single-nucleotide-polymorphism genotyping array
SPPV	Sheeppox virus
SRY	Sex-determining region Y
UMPS	Uridine monophosphate synthase
UTR	Untranslated region
VRQ	Valine–arginine–glutamine PRNP haplotype
WGS	Whole-genome sequencing
Y-chr/Y chromosome	Y chromosome
