# Supplementary material for: Inherited Disorders and Disease-Resistance Genomics in Kazakhstan Ruminants: Evidence, Limits and Breeding Priorities
Source: Int J Mol Sci. 2026 Jul 14;27(14):6268. doi: 10.3390/ijms27146268 (PMC13411912; doi:10.3390/ijms27146268)
Supplement: Supplementary file 1 [file ijms-27-06268-s001.zip › ijms-4426403-supplementary.pdf]

Table S1. Evidence-appraisal and geographic-applicability classification of retained sources

Notes: NR = not reported in the manuscript text or reference metadata available for this revision; NA = not applicable. “Exposure context” indicates pathogen molecular diagnostics retained as exposure/phenotype context, not evidence of host genetic resistance. “Method” indicates bioinformatic or population-genetic methodology used to support the review framework. The table is intended for a structured narrative review and is not a PRISMA screening table.

Reference	Species	Breed/population	Country/region of sampled animals	Evidence type	Sample size	Phenotype available	Genotype-phenotype link	Applicability to Kazakhstan	Evidence level
[1] Prime Minister of Kazakhstan, 2026	Ruminants/livestock	National livestock sector	Republic of Kazakhstan	Official policy/statistical context	NA	NA	NA	Direct national context	Context (not graded)
[2] Bureau of National Statistics, 2026	Ruminants/livestock	Cattle; sheep and goats	Republic of Kazakhstan	Official population statistics	NA	NA	NA	Direct national context	Context (not graded)
[3] FAO, 2015	Animal genetic resources	Global AnGR	International/global	Conservation and breed-resource framework	NA	NA	NA	Methodological/contextual	Context (not graded)
[4] Khamzina et al., 2024	Cattle	Native cattle breeds of Kazakhstan	Republic of Kazakhstan	Breed history; genetic characteristics	NR in manuscript	No direct disease phenotype	Indirect	Direct Kazakhstan evidence	C / context
[5] Niyazbekova et al., 2025	Cattle	Kazakh cattle	Republic of Kazakhstan	WGS; selection signatures	NR in manuscript	No direct disease phenotype	Indirect	Direct Kazakhstan evidence	C
[6] Khamzina et al., 2025	Cattle	Kazakh Whiteheaded cattle	Republic of Kazakhstan	Resequencing; ancestry; selection signatures; candidate variants	NR in manuscript	No direct disease phenotype	Indirect	Direct Kazakhstan evidence	C
[7] Beishova et al., 2022	Cattle	Two Kazakh cattle breeds	Republic of Kazakhstan	150K SNP population structure	NR in manuscript	No direct disease phenotype	Indirect	Direct Kazakhstan evidence	C
[8] Dossybayev et al., 2019	Sheep	Different Kazakh sheep breeds	Republic of Kazakhstan	Microsatellite genetic diversity	NR in manuscript	No direct disease phenotype	No	Direct Kazakhstan evidence	C / context
[9] Dossybayev et al., 2025	Sheep	Fat-tailed coarse-wooled sheep breeds	Republic of Kazakhstan	Genome-wide SNP diversity	NR in manuscript	No direct disease phenotype	No/indirect	Direct Kazakhstan evidence	C
[10] Kozhakhmet et al., 2025	Sheep	Kazakh fat-tailed coarse-wool sheep	Republic of Kazakhstan	ROH analysis; genomic characterization	NR in manuscript	No direct disease phenotype	Indirect	Direct Kazakhstan evidence	C
[11] Mukhametzharova et al., 2018	Sheep	Kazakh native sheep breeds	Republic of Kazakhstan	mtDNA characterization	NR in manuscript	No	No	Direct Kazakhstan evidence	C / context
[12] Kichamu et al., 2025	Goats	Kundyzdy, Darbaza, Shokpar, Ushterek, Kenes, Kosseit ecotypes	Republic of Kazakhstan	70K SNP; diversity; ROH; immune/adaptation candidates	NR in manuscript	No direct disease phenotype	Indirect	Direct Kazakhstan evidence	C
[13] Bekmanov and Dossybayev, 2026	Cattle; sheep; goats	Kazakhstan livestock genetic studies	Republic of Kazakhstan	Review of genetic research	NA (review)	NA	NA	Direct/contextual	Context (not graded)
[14] Yamanaka et al., 2019	Cattle	Kazakhstani native cattle	Republic of Kazakhstan	mtDNA D-loop; SRY/Y-chromosome	NR in manuscript	No	No	Direct Kazakhstan evidence	C / context
[15] Abdelmanova et al., 2021	Cattle	Local steppe cattle	Russia; Kazakhstan; Kyrgyzstan	Microsatellite diversity; museum and modern samples	NR in manuscript	No	No/indirect	Comparative regional evidence including Kazakhstan	C / context
[16] Pozharskiy et al., 2020	Sheep	Five indigenous Kazakh sheep breeds	Republic of Kazakhstan	SNP genotyping; population analysis	Five breeds; N NR in manuscript	No direct disease phenotype	No/indirect	Direct Kazakhstan evidence	C
[17] Karimov et al., 2024	Sheep	Sheep of Kazakh selection	Kazakhstan/Kazakh selection	Sequencing candidate genes for agricultural traits	NR in manuscript	Agricultural trait context	Indirect/candidate	Direct or regional Kazakhstan-relevant evidence	C
[18] Akhatayeva et al., 2025	Sheep	Kazakh fat-tailed coarse-wool sheep	Republic of Kazakhstan	GWAS for body conformation traits	NR in manuscript	Yes: conformation traits	Yes: GWAS association	Direct Kazakhstan evidence	C (trait association)
[19] Tabata et al., 2019	Goats	Kazakhstani goats	Republic of Kazakhstan	mtDNA and Y-chromosome phylogeography	NR in manuscript	No	No	Direct Kazakhstan evidence	C / context
[20] Zhumadillayev et al., 2022	Sheep	Baisary fat-tailed sheep	Republic of Kazakhstan	OvineSNP50/SNP genotyping; genome composition	NR in manuscript	No direct disease phenotype	Indirect	Direct Kazakhstan evidence	C
[21] Dossybayev et al., 2025	Sheep	Fat-tailed coarse-wooled sheep breeds	Republic of Kazakhstan	mtDNA diversity and population structure	NR in manuscript	No	No	Direct Kazakhstan evidence	C / context
[22] VanRaden et al., 2011	Cattle	Dairy cattle/Holstein	International	Harmful recessive fertility haplotypes; absence of homozygotes	NR in manuscript	Yes: fertility	Yes	International method/variant relevance to imported dairy germplasm	D
[23] Fritz et al., 2013	Cattle	Dairy cattle	International	Prenatal-death haplotypes; deleterious mutations	NR in manuscript	Yes: prenatal death/fertility	Yes	International relevance to dairy germplasm	D
[24] Pausch et al., 2015	Cattle	Cattle	International	Homozygous haplotype deficiency; reproductive and rearing success	NR in manuscript	Yes	Yes	International relevance to cattle haplotype surveillance	D
[25] Dyussekenova et al., 2025	Cattle	Holstein, Hereford, Angus, Alatau, Kazakh Whiteheaded and/or tested cattle groups	Republic of Kazakhstan	Diagnostic tests for DUMPS, BLAD, HY, OH1; carrier screening	NR in manuscript	Validated defect context; carriers/negative tests	Yes for validated variants; phenotype not always observed	Direct Kazakhstan evidence	B
[26] Charlier et al., 2008	Livestock	Livestock populations	International	SNP-based mapping and management of recessive defects	NR in manuscript	Yes: recessive defects	Yes	Methodological relevance	D / method

Reference	Species	Breed/population	Country/region of sampled animals	Evidence type	Sample size	Phenotype available	Genotype-phenotype link	Applicability to Kazakhstan	Evidence level
[27] Biscarini et al., 2016	Cattle	Cattle populations	International	SNP genotypes to identify carriers of harmful recessives	NR in manuscript	Validated carrier-identification context	Yes/indirect	Methodological relevance to Kazakhstan cattle	D / method
[28] Bosse et al., 2019	Livestock	Domesticated animals/livestock	International	Deleterious alleles; domestication, inbreeding and selection	NA (review)	NA	NA	Conceptual framework	D / method
[29] Peripolli et al., 2017	Livestock	Livestock species	International	ROH applications in livestock	NA (review)	NA	NA	Methodological relevance	D / method
[30] Ceballos et al., 2018	Multiple species	Population-genomic datasets	International	ROH and population history/trait architecture	NA (review)	NA	NA	Methodological relevance	D / method
[31] Dyussekenova et al., 2025	Cattle	Local and beef cattle breeds; Kazakh Whiteheaded, Kalmyk, Auliekol, Hereford, Santa Gertrudis	Republic of Kazakhstan	Diagnostics for hypotrichosis, dilutor and osteopetrosis	NR in manuscript	Validated defect context; carrier screening	Yes for validated variants	Direct Kazakhstan evidence	B
[32] Junussova et al., 2025	Cattle	Holstein and Jersey cows	Republic of Kazakhstan / Kazakhstan-associated study	Diagnostics for HH2 and JH1 fertility haplotypes	NR in manuscript	Fertility haplotype context	Yes/indirect via known haplotypes	Direct Kazakhstan-relevant evidence	B
[33] Nurpeissova et al., 2025	Cattle	Holstein cows and breeding bulls	Republic of Kazakhstan / Kazakhstan-associated study	IL8/IL17A reproductive markers; HH6/CVM screening	NR in manuscript	Yes: reproductive function and lethal mutation context	Partial/candidate plus validated screening	Direct Kazakhstan-relevant evidence	B/C
[34] Ussenbekov et al., 2025	Cattle	Angus, Hereford, Kazakh Whiteheaded	Republic of Kazakhstan	Screening for arachnomelia, developmental duplication, arthrogryposis multiplex	NR in manuscript	Validated defect context; carrier screening	Yes for validated variants	Direct Kazakhstan evidence	B
[35] Orkara et al., 2025	Sheep	Kazakh sheep	Kazakhstan context	Epidemiological landscape and marker-assisted selection prospects	NA or NR in manuscript	Partial; disease context	Mostly indirect/candidate	Direct/contextual Kazakhstan evidence	C / context
[36] Amandykova et al., 2023	Sheep	Kazakh meat-wool sheep	Republic of Kazakhstan	GDF9 and BMP15 genetic variation	NR in manuscript	Reproductive-gene context; phenotype limited/NR	Candidate; not validated for selection	Direct Kazakhstan evidence	C
[37] Galloway et al., 2000	Sheep	Sheep populations with BMP15 mutation	International	BMP15 causal/reproductive mutation	NR in manuscript	Yes: ovulation rate/infertility	Yes	International validation for reproductive loci	D
[38] Hanrahan et al., 2004	Sheep	Cambridge and Belclare sheep	International	GDF9/BMP15 mutations; ovulation and sterility	NR in manuscript	Yes: ovulation/sterility	Yes	International validation for reproductive loci	D
[39] Wang et al., 2020	Sheep	Chinese and Kazakhstan sheep breeds	China/Kazakhstan comparative	High-density SNP selection signatures related to prolificacy	NR in manuscript	Prolificacy context	Indirect/selection signatures	Comparative regional evidence including Kazakhstan breeds	C
[40] Mulsant et al., 2001	Sheep	Booroola Merino	International	BMPR1B mutation associated with ovulation rate	NR in manuscript	Yes: ovulation rate	Yes	International validation for reproductive loci	D
[41] Wilson et al., 2001	Sheep	Booroola sheep	International	BMPR1B/ALK-6 mutation; prolificacy	NR in manuscript	Yes: ovulation/prolificacy	Yes	International validation for reproductive loci	D
[42] Bishop and Morris, 2007	Sheep; goats	Small ruminants	International	Genetics of disease resistance	NA (review)	NA	NA	Conceptual/methodological relevance	D / method
[43] Goldmann, 2008	Ruminants	Ruminants	International	PrP genetics in TSE/scrapie	NA (review)	Yes: validated PRNP risk framework	Yes/validated framework	International relevance to PRNP preparedness	D
[44] Greenlee, 2019	Sheep; goats	Small ruminants	International	Classical and atypical scrapie update	NA (review)	Yes: scrapie context	Yes/validated framework	International relevance to PRNP preparedness	D
[45] Davies et al., 2009	Livestock	Livestock species	International	Host genetic variation in infectious-disease resistance	NA (review)	NA	NA	Methodological relevance	D / method
[46] Stear et al., 2001	Livestock	Livestock species	International	Breeding livestock for disease resistance	NA (review)	NA	NA	Methodological/conceptual relevance	D / method
[47] Ferrari et al., 2024	Goats	Goats in Lombardy	Italy	PRNP variability at goat codons	NR in manuscript	Scrapie susceptibility/resistance context	Yes/indirect via known loci	International PRNP relevance; requires Kazakhstan validation	D
[48] Pailhoux et al., 2001	Goats	Goat populations with polled/intersex phenotype	International	Deletion causing polledness/intersexuality	NR in manuscript	Yes: intersexuality/polledness	Yes	International hereditary-disorder relevance	D
[49] Lovell, 1990	Goats	Goats with beta-mannosidosis	International	Caprine beta-mannosidosis pathology	NR in manuscript	Yes: neurological/developmental disease	Yes/known disorder context	International hereditary-disorder relevance	D
[50] Zhang et al., 2024	Goats	Goat populations	International	GWAS of polled phenotype and polled intersex syndrome	NR in manuscript	Yes: PIS/polledness	Yes/GWAS	International relevance; requires Kazakhstan validation	D
[51] Daetwyler et al., 2014	Cattle	Bulls	International	WGS of 234 bulls; mapping monogenic/complex traits	234 bulls	Yes: monogenic and complex traits	Yes	International WGS reference/resource relevance	D / method
[52] Hayes and Daetwyler, 2019	Cattle	1000 Bull Genomes Project	International	Applications and outcomes of 1000 Bull Genomes	NA (review/resource)	NA	NA	International integration/resource relevance	D / method
[53] Tammen et al., 2024	Vertebrate animals	OMIA resource	International	OMIA genetic resource	NA (resource paper)	NA	NA	International database/resource	D / method

Reference	Species	Breed/population	Country/region of sampled animals	Evidence type	Sample size	Phenotype available	Genotype-phenotype link	Applicability to Kazakhstan	Evidence level
								relevance	
[54] Nicholas, 2021	Animals	OMIA resource	International	OMIA advances in animal genetics	NA (resource paper)	NA	NA	International database/resource relevance	D / method
[55] FAO, 2011	Animal genetic resources	Global AnGR	International	Molecular genetic characterization guidelines	NA (guideline)	NA	NA	Methodological/conservation relevance	D / method
[56] Nicholas and Hobbs, 2014	Non-laboratory animals	Animal genetic diseases	International	Mutation discovery for Mendelian traits	NA (review)	NA	NA	Methodological relevance	D / method
[57] Lenffer et al., 2006	Animals	OMIA resource	International	OMIA platform and NCBI integration	NA (resource paper)	NA	NA	International database/resource relevance	D / method
[58] Ferenčaković et al., 2013	Cattle	Selected cattle populations	International	Autozygosity estimates from ROH	NR in manuscript	No direct disease phenotype	Methodological	ROH-method relevance	D / method
[59] Purfield et al., 2012	Cattle	Cattle populations	International	ROH and population history in cattle	NR in manuscript	No direct disease phenotype	Methodological/indirect	ROH-method relevance	D / method
[60] Ussenbekov et al., 2026	Cattle	Kalmyk and Kazakh Whiteheaded cattle	Republic of Kazakhstan	HSP70 variability; heat-stress adaptation	NR in manuscript	Adaptation/heat-stress context	Candidate/indirect	Direct Kazakhstan evidence	C
[61] Kadyrova et al., 2024	Cattle	Cattle	Republic of Kazakhstan	Molecular characterization of Anaplasma spp.	NR in manuscript	Pathogen exposure context	No host genotype	Direct Kazakhstan pathogen-exposure evidence	Exposure context (not A-D)
[62] Ostrovskii et al., 2025	Sheep	Ewes/sheep	Southern Kazakhstan	Molecular identification of Anaplasma ovis	2553 ewes reported in manuscript	Pathogen infection phenotype/exposure	No host genotype	Direct Kazakhstan pathogen-exposure evidence	Exposure context (not A-D)
[63] Kozhayeva et al., 2025	Sheep	Sheep with neurological symptoms	Republic of Kazakhstan	Molecular prevalence of Coenurus cerebralis	NR in manuscript	Yes: neurological symptoms/pathogen	No host genotype	Direct Kazakhstan pathogen-exposure evidence	Exposure context (not A-D)
[64] Zeng et al., 2025	Livestock/ticks	Samples for piroplasms, Anaplasma, Ehrlichia	Kazakhstan	Molecular detection of tick-borne pathogens	NR in manuscript	Pathogen exposure	No host genotype	Direct Kazakhstan pathogen-exposure evidence	Exposure context (not A-D)
[65] Ostrovskii et al., 2025	Cattle	Cattle	Republic of Kazakhstan	qPCR detection and genotyping of bovine leukemia virus	NR in manuscript	Pathogen infection/exposure	No host genotype	Direct Kazakhstan pathogen-exposure evidence	Exposure context (not A-D)
[66] Yang et al., 2021	Sheep	Kazakh and Suffolk sheep	Xinjiang/China or regional Kazakh-type population	Comparative spleen transcriptome; immune-responsive genes	NR in manuscript	Immune/adaptation expression context	Indirect transcriptomic association	Comparative regional evidence; not direct Kazakhstan national evidence	C (comparative only)
[67] Kazhgaliyev et al., 2025	Cattle	Kalmyk cattle	Kazakhstan-associated cattle study	ELOVL6 and CRTC2 variation and mRNA expression; growth/carcass associations	NR in manuscript	Yes: growth/carcass/productivity	Candidate genotype/expression-phenotype link	Direct Kazakhstan-relevant evidence	C
[68] Sidikhov et al., 2026	Cattle	Cattle	West Kazakhstan	Prevalence and vectors of Theileria annulata	NR in manuscript	Pathogen exposure/disease context	No host genotype	Direct Kazakhstan pathogen-exposure evidence	Exposure context (not A-D)
[69] Omarbekova et al., 2026	Sheep	Sheep with sheeppox virus isolates	Akmola, Karaganda and Almaty regions, Kazakhstan	SPPV PCR/genome sequencing/phylogenetics	NR in manuscript	Yes: clinical/pathogen context	No host genotype	Direct Kazakhstan pathogen-exposure evidence	Exposure context (not A-D)
[70] Mamanova et al., 2026	Cattle	Cattle	Republic of Kazakhstan	Integrated BLV surveillance; diagnostic complementarity/risk zoning	NR in manuscript	Pathogen exposure/surveillance	No host genotype	Direct Kazakhstan pathogen-exposure evidence	Exposure context (not A-D)
[71] Thomsen et al., 2006	Cattle	Bovine CVM cases/lines	International	SLC35A3 mutation causing complex vertebral malformation	NR in manuscript	Yes: CVM	Yes: causal mutation	International validated defect relevant to dairy germplasm	D
[72] Charlier et al., 2012	Cattle	Bovine brachyspina cases/lines	International	FANCI deletion causing fetal death and brachyspina	NR in manuscript	Yes: fetal death/brachyspina	Yes: causal mutation	International validated defect relevant to dairy germplasm	D
[73] Shuster et al., 1992	Cattle	Holstein cattle	International	ITGB2/CD18 defect causing BLAD	NR in manuscript	Yes: leukocyte adhesion deficiency	Yes: causal mutation	International validated defect relevant to Holstein germplasm	D
[74] Beishova et al., 2022	Cattle	Kazakh cattle breeds	Republic of Kazakhstan	ROH distribution	NR in manuscript	No direct disease phenotype	Indirect	Direct Kazakhstan evidence	C
[75] Häfliger et al., 2021	Cattle	Original Braunvieh cattle	International	CNGB3 variant causing recessive achromatopsia	NR in manuscript	Yes: achromatopsia/OH1	Yes: causal variant	International validated defect relevant to Alatau/Brown Swiss ancestry	D
[76] Schwenger et al., 1993	Cattle	DUMPS cattle	International	UMPS mutation causing DUMPS	NR in manuscript	Yes: embryo loss/fertility defect	Yes: causal mutation	International validated defect relevant to dairy germplasm	D
[77] Muslimova et al., 2024	Cattle	Holstein cows	Republic of Kazakhstan / Kazakhstan-associated study	SELL, MX1 and CXCR1 genotyping for mastitis resistance	NR in manuscript	Mastitis-resistance context	Candidate association/limited	Direct Kazakhstan-relevant evidence	C
[78] Jacinto et al., 2021	Cattle	Hereford cattle	International	KRT71 loss-of-function causing congenital hypotrichosis	NR in manuscript	Yes: hypotrichosis	Yes: causal variant	International validated defect relevant to Hereford/Angus-derived germplasm	D
[79] Cole et al., 2016	Cattle	Dairy cattle	International	Effects of recessive haplotypes on yield, longevity and fertility	NR in manuscript	Yes: fertility/longevity/yield	Yes/indirect haplotype effects	International fertility-haplotype relevance	D
[80] Danecek et al., 2011	Bioinformatics	VCF/VCFtools users	International	Variant-call format and VCFtools	NA (method)	NA	NA	Bioinformatics-method relevance	Method (not A-D)

Reference	Species	Breed/population	Country/region of sampled animals	Evidence type	Sample size	Phenotype available	Genotype-phenotype link	Applicability to Kazakhstan	Evidence level
[81] Purcell et al., 2007	Bioinformatics/genetics	GWAS/linkage datasets	International	PLINK toolset	NA (method)	NA	NA	Bioinformatics-method relevance	Method (not A-D)
[82] Chang et al., 2015	Bioinformatics/genetics	Large genotype datasets	International	PLINK 2.0	NA (method)	NA	NA	Bioinformatics-method relevance	Method (not A-D)
[83] Cingolani et al., 2012	Bioinformatics	SNP annotation datasets	International	SnpEff variant annotation	NA (method)	NA	NA	Bioinformatics-method relevance	Method (not A-D)
[84] Alexander et al., 2009	Bioinformatics/population genetics	Genotype datasets	International	ADMIXTURE ancestry estimation	NA (method)	NA	NA	Bioinformatics-method relevance	Method (not A-D)
[85] Browning and Browning, 2009	Bioinformatics/statistical genetics	Large genotype datasets	International	Genotype imputation and phasing	NA (method)	NA	NA	Bioinformatics-method relevance	Method (not A-D)
[86] Szpiech and Hernandez, 2014	Bioinformatics/population genetics	Genomic datasets	International	selscan; EHH-based selection scans	NA (method)	NA	NA	Bioinformatics-method relevance	Method (not A-D)
[87] Weir and Cockerham, 1984	Population genetics	Population-structure datasets	International	F-statistics estimation	NA (method)	NA	NA	Population-genetic-method relevance	Method (not A-D)
